# Recovery of MicroRNA from Stored Bone Marrow Aspirate Slides

**Published:** 2019

**Authors:** Elaheh Sadat Ghodousi, Soheila Rahgozar

**Affiliations:** Department of Biology, Faculty of Science, University of Isfahan, Isfahan, Iran

**Keywords:** Acute lymphoblastic leukemia, Bone marrow specimens, microRNAs, miR-326

## Abstract

**Background:**

Archived bone marrow aspirate slides are almost infinite, readily available resource of biospecimens that enable retrospective molecular investigations of diseases. RNAs obtained from slides has limitations in utility because of their low quality and highly fragmented nature. MicroRNAs are small (<22 nt) noncoding RNAs with various cellular regulatory roles. Due to their small size, microRNAs are less prone to degradation and modification, therefore, can be preserved well in archived tissues.

**Methods:**

The current study investigated the efficacy of archived bone marrow aspirate slides for miRNA expression analysis in pediatric leukemia. Total RNA was isolated from air-dried unstained archived slides using High pure miRNA isolation Kit with some modifications and from fresh samples using TRizol. After cDNA synthesis, RT-qPCR was then carried out using specific hsa-miR-326 LNA primers. Finally, statistical analyses were conducted using GraphPad Prism 6 software.

**Results:**

The difference observed in miRNA expression due to disease state was far greater than the differences between archived slides and their matching fresh bone marrow specimens. In fact, the expression of archival slide smears for the miR-326 closely mimicked that of fresh-frozen tissues (0.035±0.04 *vs*. 0.03±0.04) (Mean±SD, p>0.05). Differential expression of hsa-miR-326 was detected between leukemic and non-leukemic samples from archived slides or fresh frozen bone marrows.

**Conclusion:**

The demonstration that archived bone marrow aspirate slides can be utilized for miRNA expression studies offers tremendous potential for future investigations into the role that miRNAs play in the development and long term outcome of hematologic, as well as non-hematologic diseases.

## Introduction

Most of the studies which have shown the association between miRNA expression profiling of human tumors and diagnosis, progression and prognosis have used cell culture material or fresh frozen tissue 1. Archival bone marrow slide smears have been collected throughout decades of routine histopathological examination and thus are easily available in tissue archives around the world 2. They are generally retrieved with documented clinicopathological background data. Thus, they represent a priceless source for the study of different human diseases 3. Furthermore, with the advent of high-content, high-throughput molecular genetic techniques such as qRT-PCR and microarrays, there has been huge interest in mining these archival samples as a source of biological data 4.

Interestingly, miRNAs are a class of small non-coding RNAs whose survivability and expression level in unstained bone marrow slides compared with fresh tissues are largely unknown. Having the advantage of small size and also their protein protection by RISC complex has made miRNAs less susceptible to RNA fragmentation and degradation in comparison with mRNAs, and therefore their analysis in archived specimens likely provides a more accurate replication of what would be observed in fresh samples than that of mRNA species 5–7. So, if miRNAs could be analyzed in these samples, they could gain wider acceptance as biomarkers in retrospective studies of large disease cohorts and as general diagnostic, prognostic and scientific tools 8. To date, only one other study has shown the utility of air dried bone marrow slides as a source for miRNA extraction 9. So, it is less clear whether miRNAs can be preserved, isolated, amplified, and quantified using unstained bone marrow smears.

The aim of the present study was to compare mi-RNA profiles of freshly frozen and air dried slide smear identical tissue bone marrows collected from children with Acute Lymphoblastic Leukemia (ALL) in order to identify the possible use of archived slides to extract and amplify miRNA. This may be of use to many researchers, due to the absence of concomitant biobanking of patient specimens and availability of archival samples in pathology laboratories worldwide. It is obvious that before any conclusions can be drawn, a larger study of cases and controls with more miRNAs will need to be conducted.

## Materials and Methods

### Samples

Samples used in this study are existing archived fresh bone marrow specimens and matched bone marrow films taken from Philadelphia negative children with ALL, diagnosed clinically and confirmed pathologically presented at the Sayed-ol-Shohada Hospital, Isfahan, Iran. The project was approved by the University of Isfahan review board. Samples were collected from each newly diagnosed child with full written informed parent’s consent and in accordance with the ethical protocol and standards of Sayed-ol-Shohada Hospital. A summary of patient data can be found in table 1.

**Table 1.** Primary data of the patients included in the study

**Patient characteristics**	
**Sex (number/total patients, %)**	
Male	15/27, 56.52
Female	12/27, 43.48
**ALL immunophenotype (number/total patients, %)**	
T cell linage	2/27, 7.41
Pre-B and early pre-B lineage	22/27, 81.48
Burkitt type	3/27, 11.11
**mrd+(number/total patients, %)**	
Pre-B cell	11/27, 40.74
T cell	1/27, 3.7
Burkitt type	2/27, 7.41
**mrd-(number/total patients, %)**	
Pre-B cell	11/27, 40.74
T cell	1/27, 3.7
Burkitt type	1/27, 3.7
**Relapse (number/total patients, %)**	
Pre-B cell	5/27, 18.52
T cell	1/27, 3.7
Burkitt type	1/27, 3.7
Total relapse	7/27, 25.93

ALL: Acute Lymphoblastic Leukemia, mrd: minimal residual disease determined in new case patients one year after treatment.

### RNA isolation from fresh bone marrow

Before extraction, mononuclear cells were isolated using density gradient lymphoprep (Axis-Shailed Diagnostics Ltd, Oslo, Norway) according to the manufacturer’s instruction. Total RNA including preserved miRNAs was isolated from tissue samples using TRizol reagent (Invitrogen, California, USA) according to the manufacturer’s recommendations. The extracted RNA was dissolved in 30 *μl* RNase-free water.

### RNA isolation from aspirate slides

Total RNA was isolated from air-dried unstained archived slides using the High pure miRNA isolation Kit (Roche, Switzerland) according to the manufacturer’s instructions where possible, which entails an overnight Proteinase K digestion followed by a column based kit extraction and was modified to include a slide smear scraping step.

### Reverse transcription and real-time PCR

Complementary DNA (cDNA) synthesis for miR-326 and U6 small nuclear RNA (RNU6) was carried out on 100 *ng* of total RNA, using the miR-CURY LNA^TM^ Universal RT microRNA PCR kit (Exiqon, Denmark). The tubes were incubated at 42 °*C* for 60 *min*, and then reverse transcriptase enzyme was heat-inactivated at 95 °*C* for 5 *min*. Afterward, cDNA product was subjected to real-time quantitative PCR using ExiLENT SYBR Green master mix, along with the specific locked nucleic acid (LNA) PCR primer sets (Exiqon, Denmark) on a Chromo4^TM^ system (Bio-Rad, USA). RNU6 small nucleolar RNA was quantified as internal control for data normalization. A no-reverse transcription (no-RT) control was used to detect any potential non-specific amplification of genomic DNA and No Template Controls (NTC) were run to evaluate contamination. All reactions were performed in duplicates.

### Polyacrylamide gel and T/A cloning

The specificity of primers was evaluated by running miR-326 and RNU6 real-time PCR products on 12% poly acrylamide gel to see the solo amplified band. The resultant electrophoresis bands were then T/A cloned into pTG19-T vector (Vivantis, Malaysia) and sent for sequencing.

### Statistical evaluation

All data was collected using Microsoft Excel and was statistically analyzed using GraphPad Prism 6 software. Both small RNA had PCR efficiencies nearing 100%. A Mann-Whitney test was performed for statistical analysis of the data after testing the normal distribution with Kolmogorov-Smirnov normality test (KS-test). p<0.05 was considered statistically significant. Fold change analysis was conducted using Livak method based on formulas below:

Equation 1: Where a target gene (miR-326) was measured in a test sample relative to a calibrator sample, normalized to the expression of a reference gene (RN-U6):
  $$\begin{array}{l} \Delta {\text{C}}_{\text{t}(\text{test})}={\text{C}}_{\text{t}(\text{target},\text{test})}-{\text{C}}_{\text{t}(\text{reference},\;\text{test})}\\ \Delta {\text{C}}_{\left(\text{calibrator}\right)}={\text{C}}_{\text{t}(\text{target},\;\;\text{calibrator})}-{\text{C}}_{\text{t}(\text{reference},\;\;\text{calibrator})}\\ \Delta \Delta {\text{C}}_{\text{t}}=\Delta {\text{C}}_{\text{t}(\text{test})}-\Delta {\text{C}}_{\left(\text{calibrator}\right)}\\ \text{FOLD}\;\text{CHANGE}=2^{-\Delta \Delta \text{Ct}} \end{array}$$
Equation 2: Where the effects of an experimental treatment were measured on the expression of a candidate gene (miR-326 and RNU6):
  $$\begin{array}{l} \Delta {{\text{C}}^{\prime }}_{\text{t}}={\text{C}}_{\text{t},\;\text{Treatment}\;(\text{archived}\;\text{sample})}-{\text{C}}_{\text{t},\;\text{No}\;\text{treatment}\;(\text{fresh}\;\text{sample})}\\ \text{FOLD}\;\text{CHANGE}=2^{-\Delta {\text{C}}^{\prime }\text{t}} \end{array}$$

## Results

### Polyacrylamide gel electrophoresis (PAGE)

**Figure 1.**
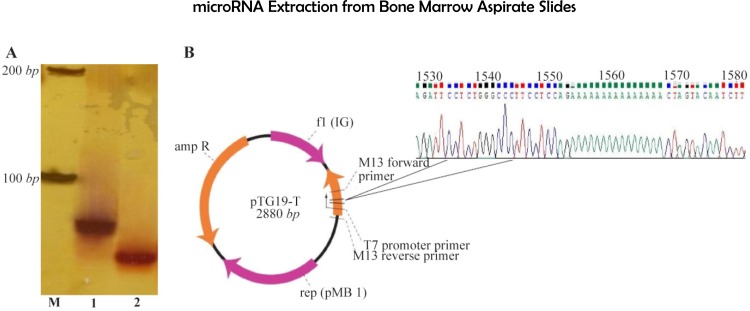
A) Polyacrylamide gel electrophoresis (12%) using 5× TBE buffer (constant voltage of 120 *V*, 90 *min*) and stained with silver nitrate solution. Unique bands showed that the miR-326 and RNU6 primers operate effectively and specifically. Lane codes: M. Ladder; 1. RNU6; 2. hsa-miR-326 B) Has-miR-326 sequencing. The resultant has-miR-326 electrophoresis band was T/A cloned into pTG19-T vector and sent for sequencing. The obtained sequence showed that the miR-326 primer operates effectively and specifically.

To determine the specificity of primers, analysis was performed on a 12% polyacrylamide gel and the gel was stained with silver nitrate solution which revealed unique bands for both miR-326 and RNU6 PCR products, comparable with the marker (Figure 1A). The result from the PAGE assay showed that the miR-326 and RNU6 primers operate effectively and specifically.

### T/A cloning and sequencing

The existence of multiple miRNA isoforms and also small size of miRNAs present a significant challenge in miRNA quantification. To further determine our mi-RNA specific detection, primer specificity was also tested using T/A cloning. The obtained sequence was aligned with has-miR-326 sequence in miRBase (MI-MAT0000756). The evenly-spaced and sharp nucleotide peaks and the lack of noise represent the accuracy of sequencing (Figure 1B).

### Archived slide samples in comparison with fresh bone marrow specimens

**Figure 2.**
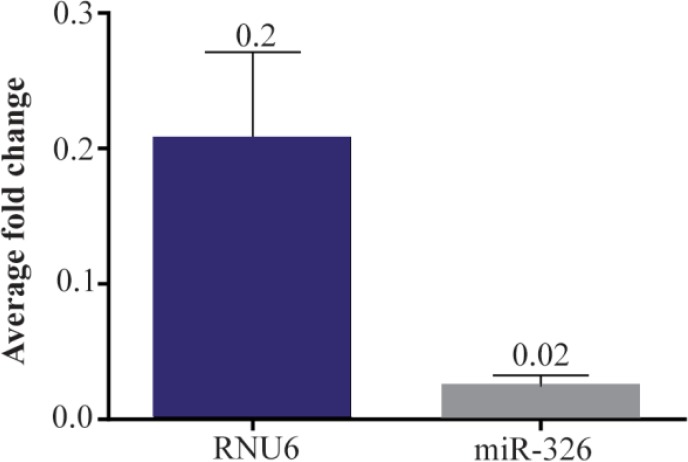
Small RNAs fold change of matched patient fresh bone marrow and archived slide samples. The overall fold change in miRNA expression in fresh bone marrow samples and their matching archived slide (based on equation 2, under “statistical evaluation”) as shown here is negligible, which means the treatment (here storage on archived slides) has negligible effect on the quantity of these RNAs.

The relationship in miRNA expression between matching archived slides and fresh frozen bone marrow samples was investigated to determine the utility of archived slides for expression analysis. Where slide sample microRNA expression was compared to its matching bone marrow (Figure 1), equation 2 was used. In fact to evaluate archived bone marrow aspirate slides for miRNA expression, twenty seven fresh bone marrow and matched unstained archived samples (Table 1) were analyzed for hsa-miR-326 expression. The average mean fold change in expression (using the Livak method 10) between fresh and matched archived samples for miR-326 and RNU6 were minimal (Figure 2, Average Fold Change).

**Figure 3.**
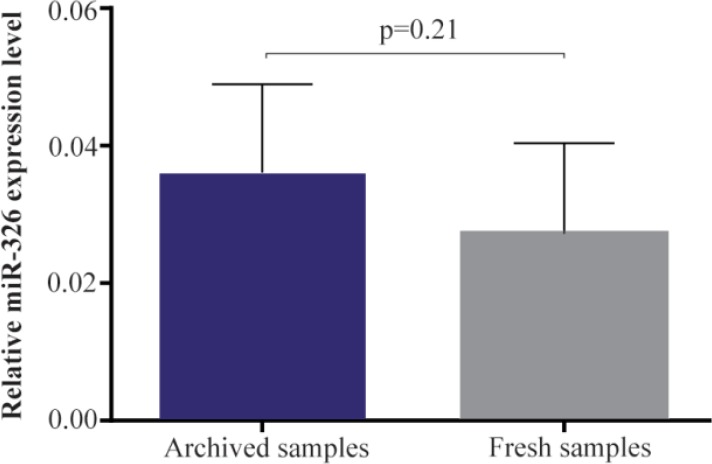
Difference in hsa-miR-326 expression in archived and fresh leukemic samples. MiR-326 expression level of archival slide smears was close to the miRNA expression level of fresh-frozen tissue and the difference was not significant (0.035±0.04 *vs.* 0.03±0.04, respectively, p-value: 0.21). In other words, the bone marrow and archived slides showed similar expression. All samples were normalized to RNU6.

To confirm the biological relevance of material extracted from archived bone marrow smears, differential miRNA expression analysis of miR-326 was performed on 27 leukemic and 13 non-leukemic samples. Where a miRNA expression difference was determined between diseased and non-diseased states (Figure 3), Equation 1 was used with normalization to RNU6.

The differences found in miRNA expression due to disease state were far greater than the differences between archived slides and their matching fresh bone marrow (p=0.21). In fact, the expression level of archival slide smears for the miR-326 was 0.035±0.04 (mean±SD; n=27), which was comparable with the miRNA expression level of fresh-frozen tissue (0.03±0.04; n=27). Thus, miRNA expression studies can be reliably performed with routinely obtained pathological materials and the results are similar to the yield from snap-frozen tissues.

## Discussion

In clinical investigations, materials such as fresh tissues, cultured cells or fresh frozen samples are seldom available which hampers the application of powerful molecular biological techniques in follow-up and retrospective studies 11. Therefore, the use of glass slide smears as a source would be very helpful. In other words, pathology and histology laboratories worldwide contain a vast stock of archived samples that can potentially be used for molecular analysis. Importantly, given the length of the storage period for these samples and their extensive clinicopathological data, retrospective examination of specific molecular markers and clinical disease association is possible 12. There are, however, little data about miRNA recovery from archival bone marrow slides.

## Conclusion

In the current study, a well-known and widely expressed miRNA, miR-326 was the main focus. To confirm the biological relevance of material extracted from archived bone marrow smears, differential miR-326 expression analysis was performed on leukemic and non-leukemic samples. Our results showed that miR-326 expression level of archival slide smears was comparable with the miRNA expression level of fresh-frozen tissue (0.035±0.04 *vs* 0.03±0.04, respectively). In addition, the average mean fold change between the fresh and matched archived samples for miR-326 and RNU6 was shown to be minimal (p=0.21). Collectively, data revealed that the accessibility of the miRNA from archival unstained bone marrow slides is comparable with fresh frozen specimens. These results may facilitate studies evaluating miRNA expression profiles by increasing the number of available samples which may be of use for diagnostic, prognostic and therapeutic purposes in hematological neoplasms. Investigating larger populations of cases and controls, ALL patients and the application of diverse miRNAs may help increase the validity of results provided in this study.
